# Decreased Absolute Number of Circulating Regulatory T Cells in Patients With Takayasu’s Arteritis

**DOI:** 10.3389/fimmu.2021.768244

**Published:** 2021-12-23

**Authors:** Wen Jia, Zi-Li Fu, Xia Wang, Jing Luo, Cheng-Lan Yan, Jian-Ping Cao, Yan-Liu, Jian-Fang Xie, Guang-Ying Liu, Chong Gao, Xiao-Feng Li

**Affiliations:** ^1^ Department of Rheumatology, The Second Hospital of Shanxi Medical University, Taiyuan, China; ^2^ Department of Rheumatology, The First Hospital of Shanxi Medical University, Taiyuan, China; ^3^ Department of Pathology, Brigham and Women’s Hospital, Harvard Medical School, Boston, MA, United States

**Keywords:** Takayasu’s arteritis, Treg cell, Th17 cell, IL-6, TNF-α

## Abstract

**Background:**

Takayasu’s arteritis (TA) is a type of primary large vessel vasculitis. Th1, Th17, and Tfh cells have been reported to be associated with TA relapse. However, the relationship between regulatory T cells (Tregs) and TA remains unclear.

**Objective:**

To analyze the levels of circulating lymphocytes, especially Treg cells (CD4^+^CD25^+^FOXP3^+^ T cells) and serum cytokines in TA patients and explore their relationship with their changes and TA disease activity.

**Methods:**

A total of 57 TA patients and 43 sex- and age-matched healthy controls (HCs) were enrolled. According to NIH standards, 36 patients had active disease status. Flow cytometry combined with counting was used to detect the absolute numbers and ratios of Th1, Th2, Th17, and Treg cells in the peripheral blood of all the subjects. Magnetic bead-based multiplex immunoassay was used to detect cytokines.

**Results:**

Compared to HCs, the absolute number and proportion of peripheral Treg cells in TA patients was significantly decreased, while Th17 cells were significantly increased. Furthermore, compared to the inactive group, the TA active group had significantly increased levels of interleukin (IL)-6, IL-10, and tumor necrosis factor (TNF)-α, but lower IL-10 levels. The absolute number of Th2 cells was negatively associated with platelet (PLT) and NIS scores in TA patients. The proportion of Th2 cells was negatively associated with the erythrocyte sedimentation rate in TA patients. After treatment, Treg cells were markedly increased.

**Conclusion:**

There was a Th17-Treg cell imbalance with a significant reduction in peripheral Treg cells and an increase in Th17 cells in TA patients compared to the HCs. The levels of IL-6, IL-10, IL-17, and TNF-α appeared to be related to disease activity.

## Introduction

Takayasu’s arteritis (TA), a primary large vessel vasculitis, causes chronic, progressive, and non-specific inflammation of the aorta and its main branches, and stenosis and occlusion of various arteries, which leads to ischemic manifestations. A variety of immune dysfunctions are involved in the occurrence and development of TA (1).

Infiltration of a variety of inflammatory cells in the blood-vessel walls is the main pathological manifestation of TA. During pathological bodily states, such as infections, dendritic cells (DCs) are activated and naïve T cells differentiate into helper T (Th) cells (namely, Th1, Th2, and Th17), which may lead to TA (2).

Recent studies have demonstrated that Th17 cells are upregulated in TA and play an important role in its pathogenesis (3, 4). Regulatory T cells (Tregs) are the key anti-inflammatory cells of the immune response. Zhang et al. (5) found that overactivation of mTORC1 in TA patients can upregulate the expression of Th1 and Th17 cells. Meanwhile, the expression of CD8^+^ Treg remained normal. However, CD4^+^CD25^+^FOX3^+^ Tregs were not studied.

This study aimed to analyze the levels of circulating lymphocyte subsets, especially Treg cells (CD4^+^CD25^+^FOXP3^+^ T cells),and cytokines in TA patients, and to explore their relationship with TA disease activity. These findings lead to novel methods of TA diagnosis and treatment.

## Materials and Methods

### Patients

A total of 57 newly diagnosed and untreated TA patients, who visited the Department of Rheumatology and Immunology of the Second Hospital of Shanxi Medical University between March 2016 and May 2019 and met the 1990 American College of Rheumatology TA classification criteria (6) were enrolled. TA patients were 18–51 years (39.05 ± 14.86 years), the male-to-female ratio was 1:5.3. We also enrolled 43 age- and sex-matched healthy adults as healthy controls (HCs) by frequency matching. Informed consent was obtained from all the participants and the study was approved by the Institutional Review Board of the Second Hospital of Shanxi Medical University.

### Clinical Indicators

ESR was measured using the Westergren method, C-reactive protein (CRP) was analyzed using immune turbidimetry. Platelets were measured using light transmission aggregometry. Magnetic bead-based multiplex immunoassays were used to detect serum interleukin (IL)-6, IL-10, IL-17, and tumor necrosis factor (TNF)-α levels. In accordance with the National Institutes of Health (NIH) standard (7), TA disease activity was evaluated on the basis of the NIS score (arteritis inactive group ≤1; TA active group: ≥2). Based on the NIS score, the disease active and inactive groups comprised 36 and 21 patients, respectively.

### CD4+ T Lymphocyte Subset Detection

**Figure 1:**
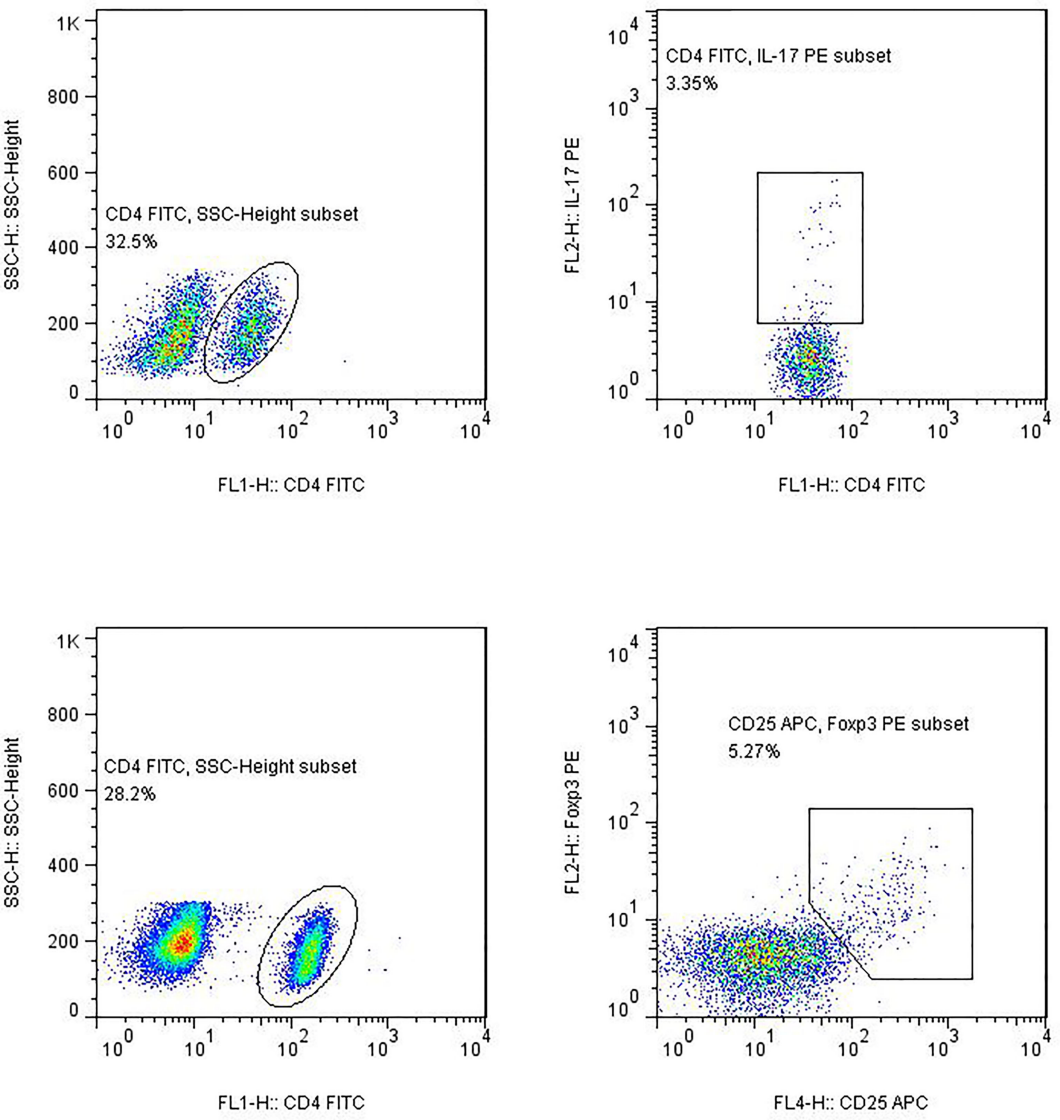
Gating for Th17 cells and Treg cells.

(1) Th1, Th2, and Th17 Cell cultures and Labeling: An 80 μl blood sample together with 10 μl phorbol myristate acetate working solution (final concentration, 30 ng/ml), 10 μl ionomycin working solution (final concentration, 750 ng/ml), and 1 μl GolgiStop was incubated at 37°C and 5% CO_2_ for 5 h. The samples were then divided into two tubes, followed by staining with anti-CD4-FITC antibodies at room temperature in the dark for 30 min. To the tubes was added 1 ml freshly prepared fixation/permeabilization solution; the tubes were then placed in an incubator at 4°C in the dark for 30 min. Anti-IL-4-PE and anti-interferon gamma (IFN-γ)-APC were added to tube A; Anti-FITC-CD4 and anti-IFN-γ-APC (intracellular staining) were used to detect Th1 cells, while anti-FITC-CD4, and anti-IL-4-PE (intracellular staining) were used to detect Th2 cells. Anti-human IL-17-PE (intracellular staining) was added to tube B for Th17-cell analysis. The two tubes of cells were stored at room temperature for 30 min in the dark and then washed with phosphate-buffered saline (PBS). The absolute numbers of CD4^+^ T lymphocyte subsets were automatically detected using BD Multitest software (BD Biosciences, Franklin Lakes, NJ, USA). All immunofluorescence antibodies were purchased from BD Biosciences.

(2) Detection of Treg Cells: Anti-CD4-FITC and anti-CD25-APC were added to an 80 μl blood sample and incubated at room temperature in the dark for 30 min. Then, 1 ml freshly prepared fixation/permeabilization solution was added to each tube, mixed, and incubated at 4°C for 30 min. AntiFOXP3-PE (intracellular staining) was added and incubated at room temperature for 30 min in the dark, followed by washing with PBS and detection of Treg cells using flow cytometry. All immunofluorescence antibodies were purchased from BD Biosciences.

(3) Flow cytometry: The stained cells were measured using flow cytometry (Calibur; BD Biosciences) within 24 h. Based on the scatter plot of the forward angular scattered light relative to the lateral angular dispersive light (side scatter (SSC)), the lymphocytes were gated to distinguish them. CD4 was used to distinguish CD4^+^ T cells from the SSC gate; 10,000 cells from the gate were taken. The relative percentages were obtained and analyzed using CellQuest software. The absolute number of cells in each subgroup was calculated using the following equation: absolute cell number = percentage of positive cells in each subset × absolute number of CD4^+^ T cells (cells/μl) cells/μl whole blood (**Figure 1**).

### Detection of Cytokine Levels by Cytometric Bead Array

The serum was separated from 4 ml of venous blood after 1–2 h and stored at −20°C. IL-6, IL-10, IL-17, and TNF-α were measured using flow cytometry. A cytometric bead array (CBA) kit was purchased from Jiangsu Sage Biotechnology Co. Ltd. (Jiangsu, China) and used according to the manufacturer’s instructions. The results were expressed as pg/ml.

### Statistical Analysis

PASS 11.0 software was used for statistical treatment for sample size. Previous studies have obtained the mean and standard deviation of each group. The sample size is 1:1, the two-sided test is 0.05, and the minimum test power is 0.8. It is calculated that the healthy control group and TA group requires 38 cases, and the active group and disease-active group requires16 cases. SPSS Statistics l24.0 software (IBM Corp., Armonk, NY, USA) was used for the statistical analysis. Data are expressed as mean ± standard deviation (mean ± SD). Normal measurement data were compared between groups using the independent sample t-test. The data distribution was tested using a non-parametric test. Normally distributed variables were analyzed using Pearson correlation analysis, while non-normally distributed variables were analyzed using Spearman correlation analysis. GraphPad (GraphPad Software Inc., San Diego, CA, USA) was used to perform receiver operating characteristic (ROC) curve analysis, to determine the accuracy of cytokine level for predicting TA disease activity. The paired t-test was used to assess the differences in variables before and after treatment. P-values <0.05 were considered significant.

## Results

### Absolute Number of Th1, Th2, Th17, and Tregs, and the Th17/Treg Ratio

The absolute numbers of Th1 cells were increased in TA (185.5 ± 145.0 *vs* 115.3 ± 66.9 cells/µl, p = 0.019) (**Figure 1**). The absolute numbers and proportion of Th17 cells were also markedly increased in TA (11.9 ± 9.3 *vs* 4.6 ± 1.6 cells/µl, p <0.001; 1.3 ± 0.9% *vs* 0.7 ± 0.3%, p <0.001). We also observed a significant decline in the absolute numbers and proportion of peripheral CD4+ Treg cells in the TA patients compared to the HCs (30.2 ± 14.2 *vs* 37.1 ± 9.2 cells/µl, p = 0.001; 3.4 ± 1.6% *vs* 5.5 ± 1.1%, p <0.001). There were no differences in the absolute numbers or proportion of Th2 cells, between the TA patients and the HCs (9.9 ± 5.9 *vs* 8.3 ± 5.0 cells/µl, p = 0.179; 1.1 ± 0.7% *vs* 1.2 ± 0.7%, p = 0.277).

**Figure 2:**
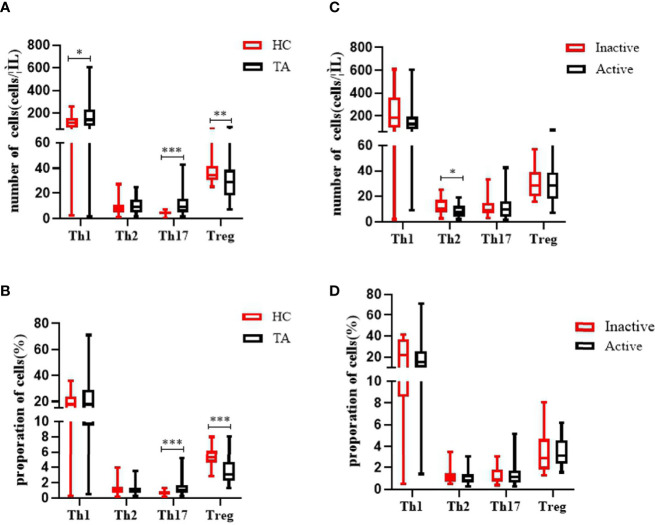
Characteristics of the absolute numbers and proportions of Th17 cells and CD4Treg cells in the PB of patients with TA. **(A, B)** The levels of Th1 cells and Th17 cells in PB were significantly increased in patients with TA (n = 57). The absolute number and the proportion of CD4Treg cells were significantly decreased in TA (n = 57). There were no significant changes of the absolute numbers and proportion of Th2 in PB between healthy controls (n = 43). **(C, D)** The absolute number of Th2 cells in PB were significantly decreased in active patients with TA (n = 36). Neither the absolute number nor proportion of Th1, Th17, and Treg cells were altered significantly between active TA patients (n = 36) and inactive TA patients (n = 21). Data were presented as mean ± SD. Shown are the significant differences assessed by the Mann–Whitney U test. *P < 0.05; **P < 0.001, ***P < 0.001. P < 0.05 was considered statistically significant. TA, Takayasu’s arteritis; PB, peripheral blood; Tregs, regulatory T cells.

Compared to the inactive TA group, we observed increased numbers of peripheral Th2 cells in the active TA group (8.4 ± 5.0 *vs* 12.3 ± 6.5 cells/µl, p = 0.026). There were no differences in the absolute numbers or proportion of Th1 cells between the groups (158.2 ± 121.8 *vs* 232.4 ± 171.0 cells/µl, p = 0.132; 19.1 ± 14.5% *vs* 22.2 ± 12.9%, p = 0.254). Neither the absolute number nor the proportion of Th17 cells was increased (12.0 ± 10.0 *vs* 11.7 ± 8.0 cells/µl, p = 0.679; 1.3 ± 0.9% *vs* 1.2 ± 0.7%, p = 0.882). Similarly, there were no significant differences in Treg cells between the active and inactive groups (30.0 ± 15.3 *vs* 30.7 ± 12.4 cells/µl, p = 0.697; 3.4 ± 1.3% *vs* 3.5 ± 2.0%, p = 0.597) (**Figure 2**).

### Il-6, TNF-α, and IL-17 Increased in Disease-Active Group

**Figure 3:**
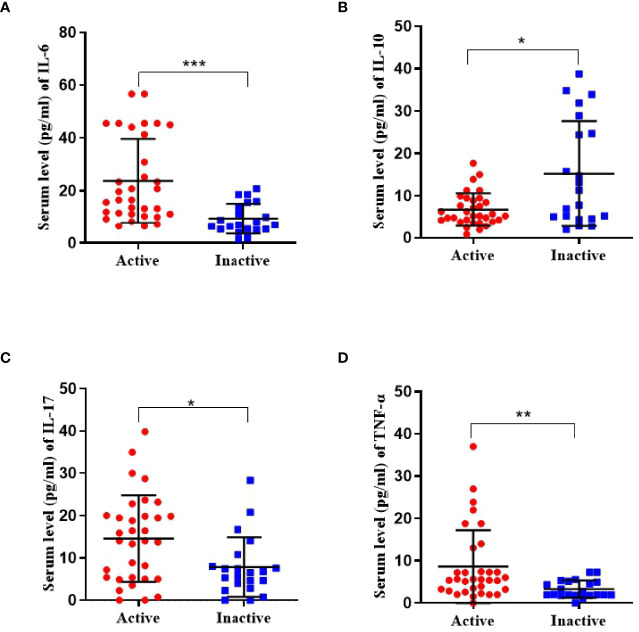
Serum concentrations of cytokine (including IL-6, IL-10, IL-17, and TNF-α) in active TA patients (n = 36) and inactive TA patients (n = 21). **(A, C, D)** The concentration of IL-6, IL-17, and TNF-α was significantly upregulated. **(B)** That of IL-10 was found reduced. Data were presented as mean ± SD. Shown significant differences are assessed by the Mann–Whitney U test. *P < 0.05; **P < 0.001, ***P < 0.001. P < 0.05 was considered statistically significant.

The active disease group had significantly higher IL-6 levels (23.7 ± 16.0 *vs* 9.4 ± 5.7 pg/ml, p <0.001), and lower IL-10 levels (6.8 ± 3.6 *vs* 15.2 ± 12.4 pg/ml, p = 0.024) compared to the inactive group. The concentrations of IL-17 (14.6 ± 10.2 *vs* 7.9 ± 7.0 pg/ml, p = 0.016) and TNF-α (8.6 ± 7.6 *vs* 3.3 ± 2.0 pg/ml, p = 0.001) (**Figure 3**).

### ROC Curve for Analysis for the Prediction of TA Disease Activity

**Figure 4:**
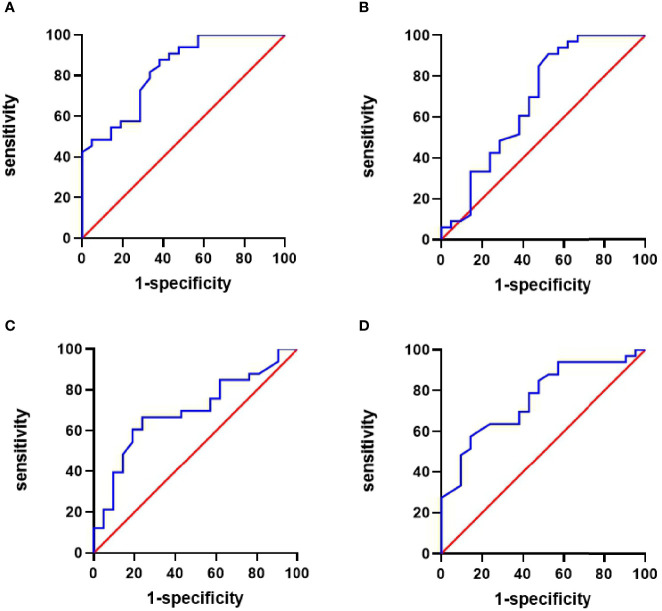
Receiver operating characteristic (ROC) curve of cytokines for predicting the activity of TA patients. **(A)** The area under the ROC curve (AUC) of IL-6 was 0.827, sensitivity 87.9%, and specificity 61.9%. **(B)** AUC of IL-10 was 0.683, sensitivity 47.6%, and specificity 90.9%. **(C)** The AUC of IL-17 was 0.696, sensitivity 66.7%, and specificity 76.2%. **(D)** AUC of TNF-α was 0.762, sensitivity 57.6%, and specificity 85.7%.

Based on the NIS scores, the areas under the ROC curve (AUCs) for IL-6, IL-10, IL-17, and TNF-α were 0.827 (sensitivity and specificity, 87.9 and 61.9%, respectively); 0.683 (47.6 and 90.9%), 0.696 (66.7 and 76.2%), and 0.762 (57.6 and 85.7%), respectively (**Figure 4**).

### The Absolute Number of Th2 cells Was Negatively Correlated With ESR and NIS

Vascular stenosis was classified as mild (score 1, <30%), moderate (score 2, 30–69%), severe (score 3, 70–99%), or occlusive (score 4, >99%). The correlations between the scores and the absolute and relative numbers of CD4^+^ T cell subsets were analyzed.

**Figure 5:**
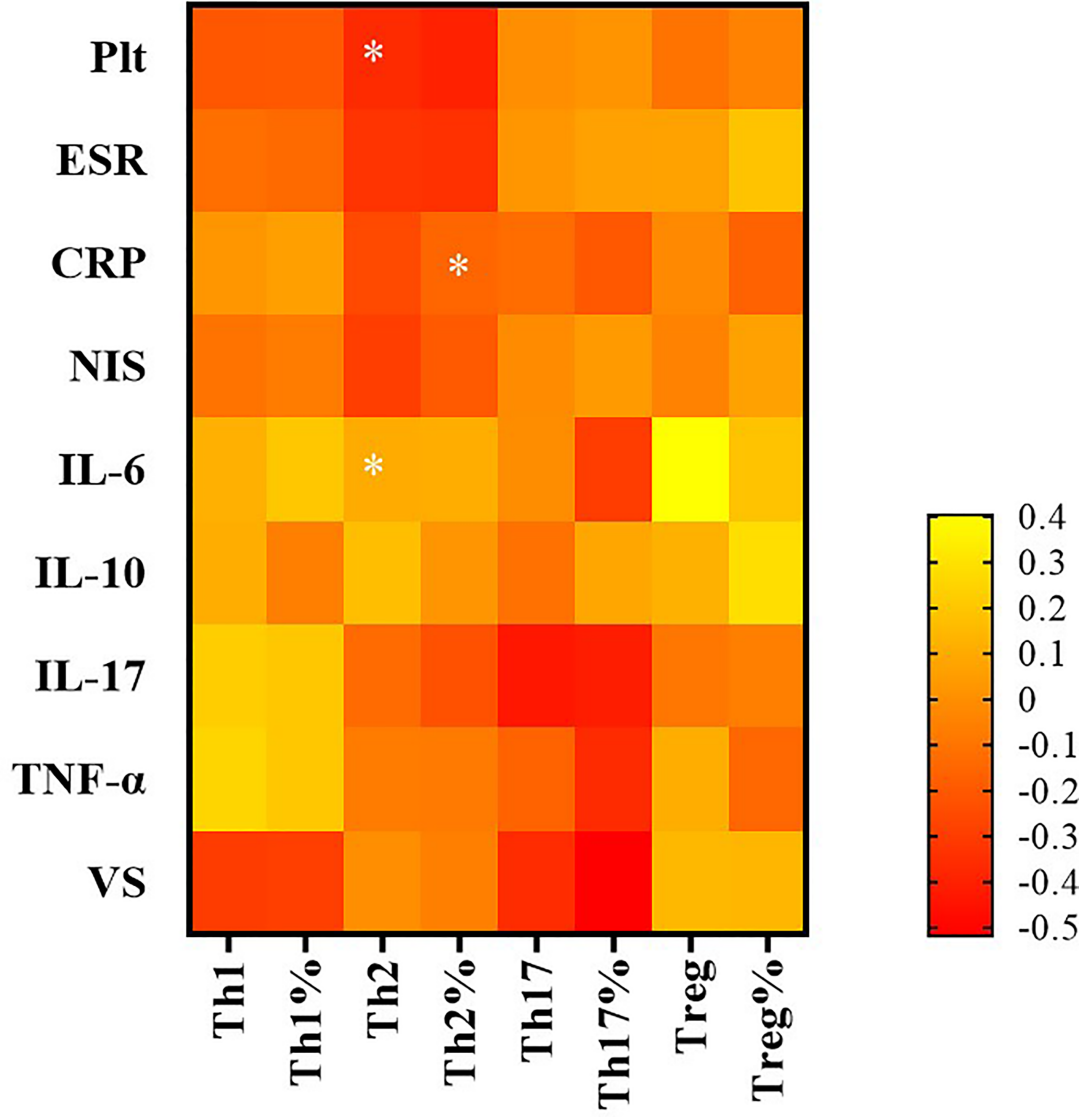
The correlation between CD4^+^ T subsets and clinical indicators by Spearman correlation test. In TA patients, that of Th2 cells was negatively associated with platelet (PLT) and NIS score; the proportion of Th2 cells was negatively associated with erythrocyte sedimentation rate (ESR). *P < 0.05; P < 0.05 was considered statistically significant.

The absolute number of Th2 cells was negatively associated with platelets (PLT), C3 and C4, and NIS scores in the TA patients (r = −0.366, p = 0.016; r = −0.390, p = 0.007; r = −0.435, p = 0.002; and r = −0.295, p = 0.047, respectively). The proportion of Th2 cells was negatively associated with the ESR in the TA patients (r = −0.342, p = 0.048). The absolute number of Th17 cells correlated negatively with C3 in TA patients (r = −0.625, p = 0.002).There was no significant correlation with PLT, IgG, IgA, IgM, C4, ESR, CRP, NIS, vascular stenosis, IL-6, IL-10, IL-17, or TNF-α (p >0.05). The proportion of Th17 cells was not significantly correlated with the levels of PLT, IgG, IgA, IgM, C3, C4, ESR, CRP, NIS, vascular stenosis, IL-6, IL-10, IL-17 or TNF-α (p >0.05). The Th17/Treg ratio and Treg cells were not significantly correlated with the levels of PLT, IgG, IgA, IgM, C3, C4, ESR, CRP, NIS, vascular stenosis, IL-6, IL-10, IL-17 or TNF-α (p >0.05) (**Figure 5** and **Table 1**).

**Table 1:** Correlation analysis.

	Th17 (cells/μl)		Th17%		Tregs (cells/μl)		Treg%	
	r	P	r	P	r	P	R	P
Plt (*109/L)	−0.007	0.966	0.011	0.942	−0.106	0.481	−0.047	0.756
ESR (mm/h)	0.020	0.900	0.061	0.696	0.066	0.661	0.184	0.222
CRP (mg/L)	−0.126	0.476	−0.201	0.255	−0.026	0.866	−0.166	0.270
NIS (score)	−0.019	0.902	0.038	0.800	−0.050	0.742	0.063	0.676
VS (score)	−0.316	0.248	−0.519	0.084	0.143	0.657	0.136	0.613
IL-6 (pg/ml)	−0.011	0.958	−0.300	0.136	0.403	0.141	0.186	0.363
IL-10 (pg/ml)	−0.113	0.583	0.079	0.701	0.118	0.565	0.282	0.162
IL-17 (pg/ml)	−0.438	0.069	−0.415	0.087	−0.094	0.711	−0.063	0.803
TNF-α (pg/ml)	−0.164	0.423	−0.364	0.068	0.110	0.594	−0.143	0.484
	Th1 (cells/μl)		Th1%		Th2 (cells/μl)		Th2%	
	r	P	r	P	r	P	r	P
Plt (*109/L)	−0.211	0.174	−0.201	0.197	−0.366	0.016*	−0.400	0.008
ESR (mm/h)	−0.118	0.505	−0.138	0.436	−0.333	0.054	−0.342	0.048*
CRP (mg/L)	0.023	0.884	0.059	0.709	−0.252	0.103	−0.152	0.332
NIS (score)	−0.104	0.491	−0.071	0.638	−0.295	0.047*	−0.196	0.191
VS (score)	0.100	0.757	−0.004	0.991	−0.118	0.715	−0.410	0.185
IL-6 (pg/ml)	0.116	0.548	0.198	0.304	0.096	0.620	0.107	0.579
IL-10 (pg/ml)	0.000	1.00	−0.062	0.749	0.168	0.384	0.017	0.929
IL-17 (pg/ml)	0.220	0.365	0.199	0.414	−0.135	0.583	−0.228	0.348
TNF-α (pg/ml)	0.248	0.195	0.200	0.299	−0.071	0.715	−0.078	0.686

By Spearman correlation test, *P <0.05. P <0.05 was considered statistically significant. VS, Vascular Stenosis.

### Treg Cell Counts Increased After Treatment

In total, 57 patients were recruited to this study, and we observed changes in various indicators of TA before and after treatment. In the patients treated with glucocorticoids and immunosuppressants, the numbers of Th17 cells showed a downward trend after treatment, but the difference was not significant. The absolute number of Treg cells after conventional treatment was significantly higher before treatment (p = 0.004) (**Table 2**).

**Table 2:** Treg cells were increased after treatment.

	Before	After	P
Th17	13.49 (8.76, 25.52)	12.44 (4.57, 28.06)	0.454
Th17%	1.69 ±0.93	1.45 ±0.68	0.384
Treg	23.96 (16.28, 39.5)	44.46 (33.67, 56.15)	0.004**
Treg%	2.3 (1.74, 4.48)	5.03 (2.31, 7.96)	0.118

Absolute numbers of CD4^+^ T subsets, especially Tregs, were significantly increased in patients after treatment. The normal distribution data were expressed as mean± standard deviation (SD)，nonnormal distribution data were expressed as median four quantile method [M (P25, P75)]. *P < 0.05; **P < 0.01. P < 0.05 was considered statistically significant.

## Discussion

TA is a chronic and non-specific full-thickness arteritis of unknown etiology. Its pathogenesis is related to genetic factors, endocrine abnormalities, immune dysfunction, and the inflammatory response of cytokines. Activated T lymphocytes promote arterial inflammation, and a variety of T lymphocyte subsets and cytokines are involved in the pathogenesis of TA (8).CD4^+^ T lymphocytes are also known as key cell participants in vasculitis (9). It is known that immune cells such as Th1 and Th17 cells and their secreted cytokines are mainly involved in the pathogenesis of TA; the role of CD4^+^ T cells of TA has always been controversial. Previous studies have suggested that Th1 and Th2 cells involved in cellular and humoral immunity play a major role in the immune system. In recent years, it has been discovered that Th17 and Treg cells from the same source but with different immune functions play an important role in autoimmune diseases. The basis of the body’s autoimmune balance is the Th17/Treg. Once the balance is broken, it will lead to the occurrence of autoimmune diseases.

Th17 cells are an important subgroup of CD4^+^ T cells, which play a major role in causing inflammation in autoimmune diseases. Excessive expression of Th17 cells and related cytokines can tend inflammatory cells to accumulate on the blood vessel wall, stimulate fibroblasts and macrophages at the same time, induce the production of a variety of inflammatory cytokines, cause repeated inflammation and endothelial damage, leading to large arteries Arterial wall thickening and thrombosis in patients with inflammation, stenosis, and occlusive disease can occur in severe cases (10). Regulatory T cells (Regulatory T cells, Treg cells) are an important subgroup of T cells, which enable the body to maintain immune tolerance and inhibit the occurrence of autoimmune diseases. After the inflammation occurs, antigen-presenting cells such as dendritic cells and macrophages are activated to induce Treg cells to reach the site of inflammation, and downregulate the expression of autoreactive T cells and inflammatory cytokines by secreting inhibitory cytokine IL-10, exerting immune tolerance to maintain the body’s own immune balance. Th17 cells and Treg cells are closely related in the differentiation process and can be transformed into each other in a specific cytokine microenvironment. When the immune system is stable, IL-10 produced by the immune system can inhibit the production of effector T cells and promote Foxp3^+^ Treg to differentiate and maintain autoimmune tolerance; when the body has an inflammatory infiltration, the activated immune system will produce IL-6 and TNF-α to promote Th17 cells to induce inflammation, while inhibiting IL-10 mediated Treg cell production. Th17/Treg balance is a key factor in the body’s own immune balance, and it plays an important role in immune defense and immune stability. The imbalance of the ratio of pro-inflammatory Th17 cells and inhibitory Treg cells mediates the occurrence and development of a variety of vascular inflammatory autoimmune diseases (11–13).

Although the pathogenesis of aortitis remains unclear, there is considerable evidence that TA occurs due to the disruption of the original immune balance. Overactivation of T cells and decreased fever of Treg cells may lead to immune imbalance. Our results do not represent the truth of the pathogenesis. But it can reflect certain trends to some extent. We demonstrated that the absolute number of circulating Treg cells was significantly lower in the TA patients, even in the inactive group, than in the HCs, which suggests that the Treg cell reduction may be involved in disease onset. Treg cells are an important T cell subgroup that maintains immune tolerance and suppresses autoimmune diseases. Downregulation of Treg cell expression is associated with a variety of autoimmune diseases (14–16). A decrease in the absolute numbers and function of Treg cells can lead to disease. However, no difference in Treg expression was found between our active and inactive groups, which suggests that disease activity may not be related to Treg cell expression.

We also observed an increase in the absolute numbers of pro-inflammatory T-cell subsets (Th1, Th2, and Th17 cells) in TA patients. The absolute numbers and proportion of Th17 cells were significantly higher in the TA patients than the HCs, consistent with the findings of Misra (3) and Saadoun (17). Although Th17 cells did not correlated with disease activity, the IL-17 levels were significantly higher in the active compared to inactive group, which suggests that IL-17 producing cells not only increased in number, but also in terms of function. However, large-sample cohort studies are required to further explore the role of Th17 and Treg cells in TA pathogenesis.

Th1 cells are one of the Th cell subgroups with pro-inflammatory effects, which can exert their inflammatory effects by secreting IFN-γ and promote cellular immunity. Previous studies have believed that Th17 cells are the main immune cells in the acute phase, while Th1 cells are the cells for chronic inflammation of blood vessels. This study did not find that Th1 cells are abnormally expressed in TA patients, which may be proportional (36/57) to the patients in the acute phase in this study. Largely related Th2 cells can secrete IL-4 and other cytokines, which play an important role in fighting parasitic infections and allergic diseases. In autoimmune diseases, Th2 cells have an inhibitory effect on inflammation, and can inhibit the differentiation of Th1 cells by secreting IL-4, thereby reducing a variety of inflammatory factors secreted by Th1 cells and inhibiting the corresponding immune response. Similar to our study, in the study of Kong et al. (10), there was no statistical difference in the proportion of Th2 cells in TA patients compared with healthy controls. Therefore, Th2 cells have no decisive role in the pathogenesis of TA. However, Th2 cells in patients with active TA were significantly reduced compared with the inactive group. Further research found that Th2 cells can predict TA disease activity: Th2 cell expression is negatively correlated with inflammatory indicators such as ESR, NIS score, and PLT, suggesting that Th2 cells have weakened anti-inflammatory effects. Lead to disease activity; still need to increase the sample size for further research.

Interestingly, we also observed higher serum levels of IL-6, IL-17, and TNF-α in active TA patients compared to inactive patients, while the levels of IL-10 were decreased. The AUC values for IL-6, IL-10, IL-17, and TNF-α, for predicting TA disease activity were 0.827, 0.683, 0.696, and 0.762, respectively, reflecting some degree of predictive value, IL-6 and IL-17 are pro-inflammatory factors secreted by Th17 cells. IL-6 can promote T cell activation and exacerbate the inflammatory response and is an important initiator of Th17 differentiation (18). Savioli et al. found that IL-6 expression in active TA patients was higher compared to those in remission (19), in agreement with our results. Refractory arteritis has been treated successfully with IL-6 antagonists (20). IL-17 is a strong inflammatory factor, that can activate T cells and stimulate endothelial cells, epithelial cells, and fibroblasts to induce inflammation in TA, and IL-17 also has a synergistic effect with TNF-α, and upregulates IL-6 expression to jointly regulate the inflammatory response (21).The increased IL-6 and IL-17 expression seen in our active TA patients suggested that the inflammatory cytokines secreted by Th17 were involved in the development of TA.

IL-10 is a typical anti-inflammatory cytokine, mainly produced by macrophages and Treg cells. It has a variety of immune regulation and inflammatory effects, and can transmit negative feedback signals, suppress immune system activation, inhibit the activation of macrophages, and reduce cytokine production by T cells. The results of this study showed that IL-10 expression, decreased with disease activity, consistent with the change in Treg-cell expression. This suggests that TA activity could be alleviated by regulating the changes in IL-10 levels and inhibiting the secretion of inflammatory factors.

Another advantage of our study was that Treg cells were labeled by anti-CD4/CD25/FoxP3 antibodies. FoxP3, regarded as the most specific marker of Treg cells, was truly important for the suppressive function of Treg cells. Our results showed that the number of Treg cells defined as CD4^+^CD25^+^FoxP3^+^ was obviously lower than those labeled by CD4^+^CD25^+^, suggesting that CD4^+^CD25^+^ T cells could not exhibit the true level of Treg cells. Moreover, we observed, in our study, the changes of the proportion and absolute number of cells were sometimes not very consistent, and the change of the percentage of one subset is not only absolutely due to changes in its cell number, but the changes in the number of other cells. Thus, proportion of cells should not completely replace the absolute number of cells to represent the cellular level.

Despite the remarkable and clinically relevant findings in this study, there are some drawbacks to our study. The number of TA patients was not large. We further need to expand the sample size to study the pathogenesis of TA.

## Conclusion

Our study suggests that the vascular inflammation seen in the TA patients is closely related to decreased absolute numbers of Treg cells, and increased Th17 cell numbers. High levels of IL-6, IL-17, and TNF-α may also contribute to disease activity. Further investigation of the causes of decreased Treg cells is required, as this may be a useful indicator for disease activity and a potential target for TA treatment. Through *in vitro* amplification or modification of Treg cells, anti-inflammatory effects may be promoted in tissues and inflammatory microenvironments to achieve control over diseases. This phenomenon may inform new strategies for immune cell therapy.

## Data Availability Statement

The original contributions presented in the study are included in the article/supplementary material. Further inquiries can be directed to the corresponding author.

## Ethics Statement

The studies involving human participants were reviewed and approved by the Department of Rheumatology, The Second Hospital of Shanxi Medical University. The patients/participants provided their written informed consent to participate in this study.

## Author Contributions

J-FX and X-FL participated in the study design. JL, Y-L and G-YL were in charge of providing data. WJ and Z-LF participated in the data collection and interpreted the data. WJ, J-PC, and C-LY performed the statistical analysis under the supervision of J-FX and X-FL. WJ wrote the draft manuscript, and J-FX and CG helped to revise the manuscript. All authors contributed to the article and approved the submitted version.

## Funding

This project was supported by the Key R&D Project of Shanxi Province (201803D31127).

## Conflict of Interest

The authors declare that the research was conducted in the absence of any commercial or financial relationships that could be construed as a potential conflict of interest.

## Publisher’s Note

All claims expressed in this article are solely those of the authors and do not necessarily represent those of their affiliated organizations, or those of the publisher, the editors and the reviewers. Any product that may be evaluated in this article, or claim that may be made by its manufacturer, is not guaranteed or endorsed by the publisher.
